# The many fates of tissue regeneration

**DOI:** 10.1371/journal.pgen.1007728

**Published:** 2018-11-21

**Authors:** Christopher Abdullah, Robert J. Duronio

**Affiliations:** 1 Integrative Program for Biological and Genome Sciences, The University of North Carolina at Chapel Hill, Chapel Hill, North Carolina, United States of America; 2 SPIRE Postdoctoral Fellowship Program, The University of North Carolina at Chapel Hill, Chapel Hill, North Carolina, United States of America; 3 Lineberger Comprehensive Cancer Center, The University of North Carolina at Chapel Hill, Chapel Hill, North Carolina, United States of America; 4 Department of Genetics, The University of North Carolina at Chapel Hill, Chapel Hill, North Carolina, United States of America; 5 Department of Biology, The University of North Carolina at Chapel Hill, Chapel Hill, North Carolina, United States of America; Geisel School of Medicine at Dartmouth, UNITED STATES

Multicellular organisms have evolved mechanisms to repair or regenerate damaged tissues, a process important for normal development and homeostasis. The capacity for tissue regeneration can be impressive. A familiar example is whole-limb regeneration in amphibians [1], but even the mammalian liver fully regenerates after removal of two-thirds of its mass [2–4]. Tissue regeneration often occurs via activation of resident stem cell populations [5–7] but can also occur in tissues lacking niche-localized stem cells [2–4]. A major question regarding such dramatic examples of regeneration is how diverse cell types that comprise a complex organ get replenished, particularly when the tissue lacks an obvious resident stem cell population. In this issue of *PLOS Genetics*, Verghese and Su [8] address this question using an elegant paradigm for tissue regeneration in *Drosophila*, showing that stem-cell–like behavior can be induced in response to tissue damage resulting from ionizing radiation (IR).

## Cell identity changes during regeneration

Experimentally, tissue regeneration has been interrogated in a variety of model systems using either physical (e.g., surgical removal of cells or appendages, killing cells with IR) or genetic (e.g., triggering cell death by expression of pro-apoptotic genes) cell ablation to injure tissue [9, 10]. The wing imaginal disc of *Drosophila* larvae has become a staple of such studies [11–15]. This single layered epithelium, which expands from 50 to 50,000 cells during larval development, contains precursors of the adult wing and thorax and has a remarkable regenerative capacity. It can be fully restored even after IR-induced killing of half of its cells [10, 12, 13]. How does this happen, particularly when this tissue does not have a resident stem cell population poised to generate diverse cell types?

Verghese and Su addressed this question by combining IR-induced tissue damage with sophisticated cell and lineage tracking tools available in *Drosophila*, to identify the wing disc cells that contribute to regeneration and to monitor their behavior after irradiation. The authors previously identified cells in the hinge region that contribute to regeneration of the wing pouch, which ultimately becomes the adult wing blade [12, 13]. These experiments are consistent with others using genetic ablation to induce apoptosis in the pouch region of the wing disc [16]. Verghese and Su further demonstrated that after IR treatment, cells acquired the ability to proliferate and translocate from their original positions to regenerate damaged tissue [12, 13]. In addition, the authors sometimes observed the formation of ectopic wings after IR treatment.

**Fig 1:**
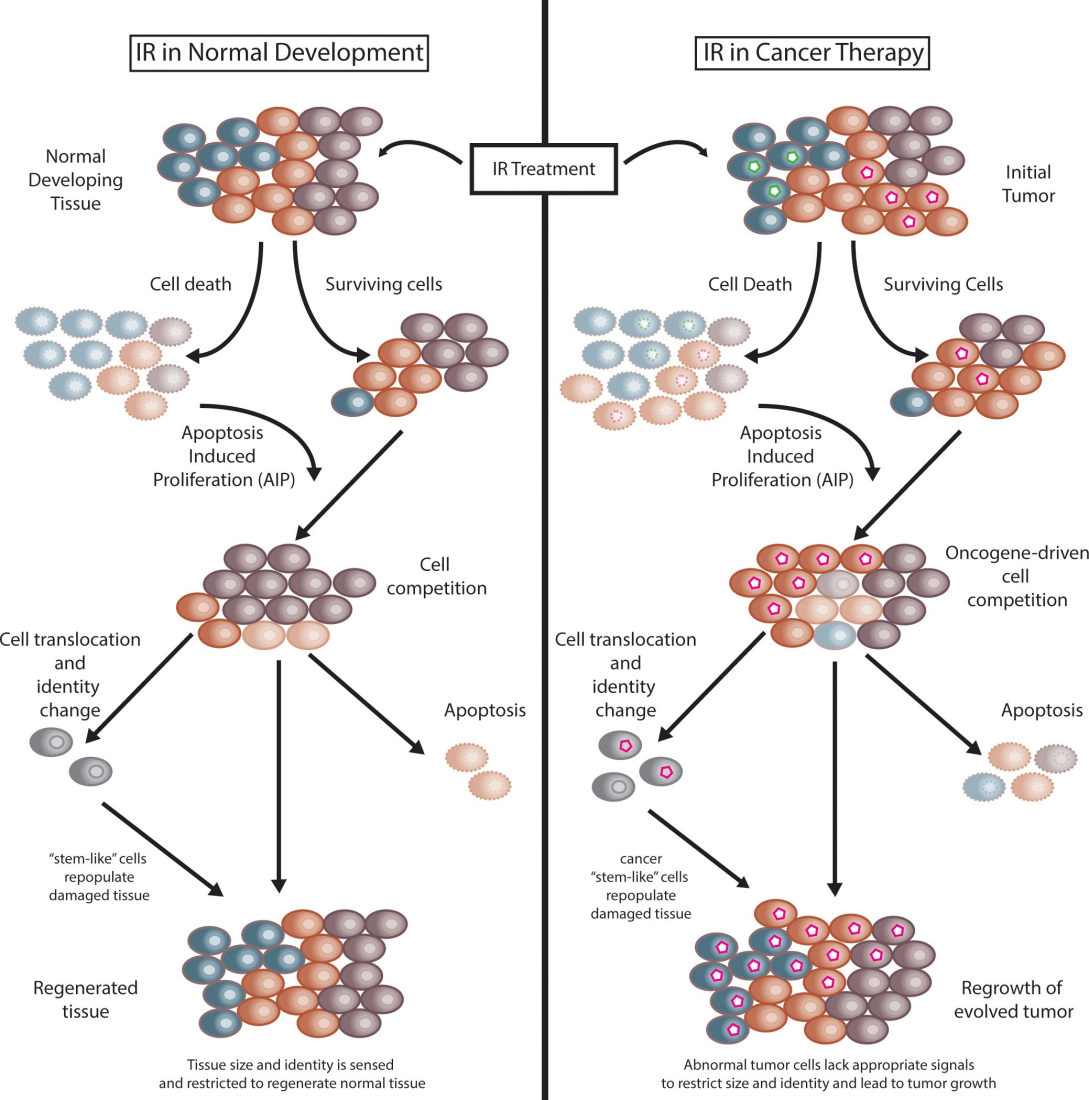
The effects of IR in normal tissue regeneration and in cancer therapy. IR is used to treat tumors in human cancers but can also be utilized to model tissue damage and regeneration after injury. (Left) In normal tissues consisting of diverse cell types (depicted as differently colored cells), IR induces cell death, and AIP can stimulate surviving cells to repopulate the damaged tissues. As cells proliferate, cell competition between pools with different proliferative capacities may occur. As a consequence, cells may contribute via different mechanisms to regenerate tissues, including proliferating to replace dead cells, undergoing apoptosis, and translocating and changing identity (gray cells) to fully regenerate all cell types in the tissue. (Right) Similar pathways may be activated after IR treatment of tumors. However, genetic changes (green or pink hexagonal nuclei) in cancer cells abnormally increase proliferative capacity and suppress apoptosis. These properties can lead to oncogene-driven cell competition within the tumor mass after irradiation, leading to expansion of cancer cell pools. Attempts to regenerate the tissue, however, are deregulated as oncogenes can drive overproliferation of tumor cells, evade apoptotic signals, and generate cancer stem-like populations of cells (gray cells with pink hexagons) that can regain proliferative capacity. Ultimately, the newly evolved tumor lacks signals that would restrict growth and normal development and will be genetically and biochemically distinct from the initial IR-treated tumor. AIP, apoptosis-induced proliferation; IR, ionizing radiation.

In their new study, Verghese and Su identify pools of differentiated cells—in addition to those in the hinge—that after IR treatment can change identity, translocate within the tissue, and contribute to ectopic disc formation (Fig 1, left). The authors suggest that differentiated cells can revert to a more “stem-like” state as a means of regenerating IR-damaged tissue. This and other studies suggest that the specific type or method of injury may directly impact the regenerative potential of the tissue. For instance, ablation of notum cells within the wing imaginal disc via genetic induction of apoptosis does not result in regeneration of the wing imaginal disc [11] in contrast to findings using IR-induced tissue damage [12, 13]. In addition to the method of damage, the original identity of the lost cells also dictates which cell types can be regenerated. For instance, genetic ablation of either notum [11] or pouch [16] cells resulted in regeneration only of the original ablated cell type (i.e., notum or pouch, respectively) rather than different types of wing disc cells. In contrast, methods that damage multiple cell types, including IR [12, 13] or genetic ablation combined with genetic depletion of c-terminal binding protein (CtBP) [17], result in the regeneration of tissue containing each of the original damaged cells and are often accompanied by the formation of ectopic wing tissue. Therefore, both the method of injury and injured cell type influence the regenerative capacity of the damaged tissue. These concepts hold true in other *Drosophila* tissues [18] as well as diverse tissues (e.g., lung, kidney, and fin) in other organisms, including human, mouse, and zebrafish [19–21].

## Tissue repair signaling

Verghese and Su also sought to identify the signals involved in changing cell identity and recruiting cells to damaged areas, which remain incompletely understood. An emerging theme is the activation of growth-promoting developmental pathways via jun nuclear kinase (JNK)-mediated stress-responsive signaling [22]. A recent study indicates that the interplay between Janus kinase/signal transducer and activator of transcription (JAK/STAT) and JNK signaling modulates the plasticity of cell identity necessary for wing imaginal disc regeneration after ablating cells by expressing a pro-apoptotic gene [17]. Additionally, this study revealed that not every apoptosis-promoting gene results in the same regenerative response even when expressed in the same cell population, a phenomenon that may be related to level of JNK signaling activated during the injury response [17]. Verghese and Su extend these findings by showing that the zinc finger homeodomain 2 (Zfh2) transcription factor involved in JAK/STAT signaling is required for the regenerative capability of certain wing disc cells after IR.

Verghese and Su also report that IR-induced caspase activity is necessary for regeneration. Previous studies demonstrated that activated caspases can drive apoptosis in wing discs and that cell death promotes apoptosis-induced proliferation (AIP) in surviving cells via an extrinsic proliferation signal [23–26]. AIP can explain how a damaged tissue could recover the lost cell numbers but does not necessarily explain how cells might change identity and/or revert to a more stem-like state. Verghese and Su provide evidence that cell death–independent caspase function might trigger such an event, but the mechanism remains unknown. Nonapoptotic roles of effector caspases have recently been identified in *Drosophila* and other organisms [25, 27–29].

How different cell lineages within damaged tissue balance their proliferative potential is also an important aspect of regeneration. Classic studies in *Drosophila* imaginal discs [30, 31] revealed that cells with different proliferative capacities compete with each other locally within a tissue. Cells with high proliferative capacity (e.g., overexpressing the Myc oncogene) can kill neighboring cells with lower proliferative capacity [32, 33]. Cell death could lead AIP to further promote proliferation of regenerative cells. Although this model (Fig 1, left) can help to explain how to repopulate the cellular mass lost after injury, questions remain as to how damaged tissues can sense and potentially restrict the size and cell identities of the regenerated tissues.

## Lessons for cancer treatment

IR-induced cell killing is often part of the treatment regimen for cancer patients and causes tissue damage that is likely subject to many aspects of regeneration discussed here. However, due to oncogene activation and/or inhibition of tumor-suppressive pathways, cancer cells rely on vastly different genetic and biochemical programs than during normal development. Tumor microenvironments are complex—heterogenous mixtures of normal and neoplastic cells—and factors such as the injury type, identity of the lost cells, and tissue microenvironment may largely dictate the extent of tissue repair. Additionally, suppression of cell death pathways in cancer cells may also lead to incomplete or erroneous activation of caspase signaling, which may affect both cell proliferation and cell identity. Therefore, IR treatment of tumors may result in unanticipated or undesired consequences (Fig 1, right). Indeed, as Verghese and Su note, increasing evidence suggests that IR treatment can induce tumor cells to dedifferentiate into a more plastic stem-like cell, which can lead to tumor relapse [34, 35]. Therefore, understanding that tumors and normal tissues respond differently to IR as well as understanding the unique biology of individual tumors are both important considerations whenever treating tumors with IR. Determining the molecular basis for these evolutionarily conserved responses may prove very informative in this regard.
